# Alcohol-induced psychotic disorder: a study of hospitalized patients in Lisbon

**DOI:** 10.1192/j.eurpsy.2022.331

**Published:** 2022-09-01

**Authors:** V. Nogueira, I. Pereira, M. Moreno, J. Teixeira

**Affiliations:** 1 Avenida do Brasil, número 53, Serviço De Alcoologia E Novas Dependências, Lisboa, Portugal; 2 Centro Hospitalar Psiquiátrico de Lisboa, Serviço De Alcoologia E Novas Dependências, Lisbon, Portugal; 3 Centro Hospitalar Psiquiátrico de Lisboa, Clínica 5, Lisbon, Portugal

**Keywords:** anti-psychotic treatment, alcohol-induced psychotic disorder, alcohol hallucinosis, alcohol-withdrawal

## Abstract

**Introduction:**

While alcohol-induced psychotic disorder (AIPD) is a well-recognised clinical disorder, relativery little is known about aspects such as epidemiology, course and treatment of the condition. Current evidence suggests AIPD can be clinically distinguised from alcohol-withdrawal delirium and schizophrenia. AIPD is associated with high comorbidity with other psychiatric disorders, high re-hospitalization and mortality rate, namely suicidal behaviour.

**Objectives:**

The objetive of the study was to examine the correlates, clinical features, psycopathology, and short-term response in an inpatient sample with alcohol-induced psychotic disorder, predominant hallucinations (ICD-10 F10.52) admitted to Centro Hospitalar Psiquiátrico de Lisboa.

**Methods:**

We collected retrospectively data from all admitted patients to our Alcohol Unit between January 2010 and January 2020 with the diagnosis of AIPD. The exclusion criteria were: presence of preexisting psychotic disorder, delirium, or other substance use disorders. We characterized our sample in Demographic categories, Clinical categories, Treatment and Short-term course.

**Results:**

A total of 113 subjects were included in the study. The prevalance of alcoholic hallucinosis was found to be 1.3% of all patients who received inpatient treatment. Most individuals reported auditory hallucinations, that iniciated when they decrease their alcohol intake, and 1 in 4 had past episodes of AIPD.

**Conclusions:**

There are specific challenges in studiyng AIPD, such as the relatively rarity of the disorder, its often transient nature and high levels of comorbidity. A high degree of recurrence and morbidity indicates a need to prevent, and intervene early with an abstinent-oriented management goal.

**Disclosure:**

No significant relationships.

